# Evaluation of oxidative markers in women with invasive cervical cancer in Lagos, Nigeria

**DOI:** 10.3332/ecancer.2021.1266

**Published:** 2021-07-15

**Authors:** Juliet O Offor, Kehinde S Okunade, Bamidele A Iwalokun, Ayodeji A Oluwole, Rose I Anorlu

**Affiliations:** 1 Department of Obstetrics & Gynaecology, Federal Medical Centre, Jabi, Abuja FCT, Nigeria; 2 Department of Obstetrics & Gynaecology, College of Medicine, University of Lagos, PMB 12003, Idi-Araba, Lagos, Nigeria; 3 Department of Molecular Biology & Biotechnology, Nigerian Institute of Medical Research (NIMR), Yaba, Lagos, Nigeria

**Keywords:** carcinoma, cervix, glutathione, malondialdehyde, vitamin C

## Abstract

Epidemiological studies have showed that low levels of antioxidants induce the generation of free radicals leading to DNA damage and further mutations seen in cancer. This study evaluated the effects of oxidative markers on the occurrence and severity of cervical cancer at the Lagos University Teaching Hospital. This was an analytical cross-sectional study carried out among women with histological diagnosis of invasive cervical cancer and their healthy cancer-free comparison group. Venous blood samples were collected from each participant for measurements of antioxidants (erythrocyte glutathione and vitamin C) and malondialdehyde (a marker of lipid peroxidation). Descriptive statistics were carried out for relevant demographic and clinical data. Associations between continuous variables were tested using the independent sample t-test or the analysis of variance for normally distributed data or the Mann–Whitney U test for skewed data, whereas categorical variables were compared using the χ^2^ test. *p* < 0.05 was considered statistically significant. The mean level of malondialdehyde (MDA) was statistically higher in women with cervical cancer than in their cancer-free counterparts (*p* = 0.032). However, the mean glutathione (32.6 ± 6.2 versus 14.2 ± 6.1 mg/dL; *p* = 0.019) and vitamin C (12.4 ± 2.3 versus 14.6 ± 2.4 µmol/L; *p* = 0.001) levels were significantly lower in the case group compared to the cancer-free group. There are statistically increasing mean levels of MDA (*p* = 0.017) and decreasing mean levels of vitamin C (*p* = 0.004) with increasing stages of the disease. This study showed that women with cervical cancer have low levels of antioxidants and an increased level of the oxidative marker. The levels of these markers become more pronounced as the disease progresses. This will, therefore, form the basis for the conduct of future randomised controlled trials of antioxidant supplementations among cervical cancer patients in sub-Saharan Africa.

## Introduction

Cervical cancer is one of the challenges encountered by women as a consequence to their sexuality and childbearing potential. It is the most common female genital tract malignancy in most developing countries [1] and it is the second most common cancer among women [2] and it ranks fourth for both mortality and morbidity in women globally [3]. About 85% of the global burden occurs in the less developed regions, where it accounts for almost 12% of all female malignancies [2, 4]. The lifetime risk of a woman developing cervical cancer in the low-resourced setting is 2%–4% [5, 6]. About 80%–90% of women with cervical cancer in developing countries present with stage III and IV of the disease [7]. The primary aetiology of the disease is persistent or chronic infection with the high-risk human papillomavirus (HPV), a sexually transmitted infection [8]. However, persistent HPV infection alone is not sufficient to cause cervical cancer. Certain cofactors, such as smoking, low socio-economic status, long-term oral contraceptive use, high parity, nutritional status, co-infection with other sexually transmitted diseases, HIV and immunosuppression, have been shown to modulate the oncogenic potential of HPV [2, 9, 10]. The nutritional aetiology of cervical cancer includes low dietary intake of vitamin C, carotenoids, vitamin E and folate [11]. Reactive oxygen species (ROS) generated from biochemical reactions *in vitro* cause lipid peroxidation products, which have been shown to induce a variety of genetic alterations, including DNA strand breaks, chromosomal abnormalities, oxidative base modifications and cellular transformation, that could result in a mutagenic lesion leading to malignant transformation [12]. The deleterious effects of these free radicals (malondialdehyde, hydrogen peroxide_,_ superoxide and nitric oxide) are counteracted by the enzymatic (superoxide dismutase, glutathione peroxidase and catalase) and non-enzymatic (α-tocopherol, ascorbic acid and reduced/oxidised glutathione) antioxidants [13]. An imbalance between the production and detoxification of free radicals results in oxidative stress that damages the proteins, lipids and DNA [13]. Many epidemiology studies have further revealed that the low levels of antioxidants induce the generation of these free radicals, leading to DNA damage and further mutations [11]. Various dietary antioxidants have shown considerable promise as effective agents for cancer prevention by reducing oxidative stress which has been implicated in the development of many diseases, including cancer [14]. Vitamin C is a water-soluble antioxidant that scavenges cancer-causing free radicals such as hydrogen peroxide to prevent lipid peroxidation and destruction of cells and also neutralises carcinogenic chemicals such as nitrosamine and nitrites [15]. Glutathione provides a primary defence against oxidative stress by its ability to scavenge free radicals or participate in the reduction of hydrogen peroxide (H_2_O_2_) and this is central to the detoxification of ROS [16]. Epidemiological studies have also demonstrated an association between antioxidants and cervical cancer [11, 17–19]; however, there is a paucity of studies on this subject in our environment. Moreover, the predominantly low socio-economic and poor nutritional factors could make these interactions different in our settings. This study, therefore, evaluated the role of antioxidant and oxidative markers in the occurrence and severity of invasive cervical cancer among women in Lagos, Nigeria.

## Patients and methods

### Study design and setting

This was an analytical cross-sectional study carried out at the Gynaecological Oncology clinic inpatient wards and the Radiotherapy Department of the Lagos University Teaching Hospital (LUTH) between August 1, 2018, and July 31, 2019. LUTH is the teaching hospital of the College of Medicine of the University of Lagos and is one of the main referral tertiary hospitals with a well-established gynaecological oncology unit in the Lagos metropolis. It is one of the centres with a functioning radiotherapy unit in Nigeria and it receives many referrals from Lagos and its surrounding states and neighbouring countries.

### Study population

We recruited women with histologic-confirmed cervical cancer who were attending the gynaecological oncology units and radiotherapy clinics of LUTH (case group) and their aged-matched cancer-free women who were attending the gynaecological clinics of the hospital for non-cancerous complaints during the period of study (comparison group). Included in the study were women aged between 25 and 65 years who had not undergone any previous treatment for any cancer. Women who were athletes, smokers, alcohol consumers, those who had undergone hysterectomy, those who had other concurrent diseases such as diabetes mellitus, renal disease, liver disease, hypertension, pulmonary disease and those who withdrew consent in the course of the study were excluded.

### Sample size estimation

The minimum sample size (*N*) was calculated using the sample size formula for comparing two means [20]:

$$N=\frac{4\sigma^{2}(Z_{\mathrm{crit}}+Z_{\mathrm{pwr}}{)}^{2}}{D^{2}}$$

where *Z*_crit_ is the standard normal deviate corresponding to the desired type I error rate (1.96) and *Z*_pwr_ is the standard normal deviate corresponding to the desired statistical power of 80% (0.842). The mean vitamin C levels in cervical cancer (1.17 ± 0.06 mg%) and their control (1.32 ± 0.08 mg%) groups were obtained from the published study by Naidu et al [4], with a minimum expected difference between the two means (*D*) being 0.15 and the standard deviation (*σ*) of 0.14. Using a 10% non-response rate, we calculated a sample size of 240, and therefore, we recruited 120 cervical cancer cases and an equal number of cancer-free controls (with normal Pap smears) into the study.

### Participants’ recruitment and data collection

Eligible participants were recruited into the study using the consecutive sampling technique. A well-structured interviewer-administered questionnaire was used by the investigators to obtain participants’ socio-demographic and clinical information such as age, parity, educational level, marital status, the presence of risk factors for cervical cancer and the International Federation of Gynaecology and Obstetrics (FIGO) stages for those with a histological diagnosis of invasive cervical cancer. Subsequently, 10 mL of venous blood samples were collected from each participant under aseptic conditions by venipuncture. A 5 mL aliquot was put into a plain sample bottle and the second 5 mL aliquot was put into a lithium heparin sample bottle. Both bottles were labelled with the participant’s code and then transported to the Biochemistry and Nutrition Laboratory of the Nigerian Institute of Medical Research for analysis and measurements of serum vitamin C, glutathione (GSH) and malondialdehyde (MDA).

### Laboratory analyses

Blood samples were centrifuged at 3,000 rpm for 10 minutes and immediately stored at −20°C before final analysis. The prepared serum was used for vitamin C and MDA estimation, while the haemolysate was used for the determination of reduced GSH.

- **Lipid peroxidation:** MDA was estimated by measurement of thiobarbituric acid reactive substances in plasma by Niehaus and Samuelsson’s [21] method. About 0.1 mL of the serum sample was mixed with 1.9 mL of the 1:1:1 ratio thiobarbituric acid (TBA)–trichloroacetic acid (TCA)–hydrogen chloride (HCl) reagent (0.37% TBA; 15% TCA; and 0.25 N HCl) making 2.0 mL. This mixture was placed in a water bath (100°C) for 15 min, and then cooled and centrifuged at room temperature (at 1000 rpm for 15 minutes). The absorbance of clear supernatant was measured against the reference blank at 535 nm.
- **Vitamin C:** This was estimated by Roe and Kuether’s method [22]. About 0.5 mL of the serum sample was mixed with 1.5 mL of 6% TCA and then centrifuged. About 0.5 mL volume of 0.5% oxalic acid was added to the supernatant fluid and then filtered. The filtrate was treated with 0.5 mL of 2,4-dinitrophenylhydrazine (2,4 DNPH) and incubated at 37°C for 3 hours and then 0.1 mL of 85% sulphuric acid (H_2_SO_4_) was added and incubated for 30 minutes. The colour developed was read at 540 nm against a blank solution containing 0.5% oxalic acid, 2,4 DNPH and water. The concentration of vitamin C in the sample was determined by extrapolation from the standard concentration–absorbance curve of the working standard vitamin C solution (0.2–2 mg/mL in 0.5% oxalic acid).
- **Reduce GSH:** This was determined by Ellman’s method [23]. One mL of 10% TCA was added to 1.0 mL homogenate and then centrifuged at 10,000 rpm for 10 minutes. One mL of supernatant was treated with 0.5 mL of Ellman’s reagent (19.8 mg of 5, 5ʹ-dithiobis-(2-nitrobenzoic acid) or DTNB in 0.1% sodium nitrate) and 3.0 mL of phosphate buffer (0.2 M, pH 8.0). The absorbance was read at 412 nm using molar absorptivity of 1.36 × 104 M cm^−1^. The concentration of GSH is calculated as follows: Volume sample/Total volume reaction × molar absorptivity.

### Statistical analysis

Data analyses were carried out using Epi Info Version 3.4.3 statistical software package (manufactured in 2007). Quantitative data were tested for normality with the Kolmogorov–Smirnov test with Lilliefors’ significance correction. Demographic and clinical data were summarised using descriptive statistics expressed as mean (standard deviation) for continuous variables and proportion (or percentages) for categorical variables. Associations between continuous variables were tested using the independent sample *t-*test or the analysis of variance (normal distribution) or the Mann–Whitney U test (skewed data), whereas categorical variables were compared using the *χ*^2^ test. The linear relationships between the oxidant and antioxidant markers levels were also tested and the strengths of these associations were determined using Pearson’s correlation coefficient and simple linear regression. *p* < 0.05 was considered statistically significant.

### Ethical considerations

Ethical approval was obtained from the study hospital’s Health Research Ethics Committee before participants’ recruitment and ethical principles of the Helsinki Declaration were applied throughout the study. All participants were counselled before their enrolment, and they read and signed an informed consent form. The investigators ensured strict confidentiality of participant information.

## Results

The overall mean age of the participants was 47.8 ± 7.0 years. There were statistically significant differences in parity (*p* = 0.002), age at first delivery (*p* = 0.001), educational level (*p* = 0.001) and marital status (*p* = 0.038) between the two groups of participants. There were no differences in participants’ age (*p* = 0.170), body mass index (BMI) (*p* = 0.132), age at coitarche (*p* = 0.129), number of lifetime sex partners (*p* = 0.485) and religion (*p* = 0.141) between the two groups of participants (Table 1).

As shown in Table 2, the mean serum MDA level was significantly higher in women with invasive cervical cancer than in their control counterparts (10.0 ± 3.0 versus 4.4 ± 1.1 nmol/mL; *p* = 0.032). However, the mean glutathione (32.6 ± 6.2 versus 14.2 ± 6.1 mg/dL; *p* = 0.019) and vitamin C (12.4 ± 2.3 versus 14.6 ± 2.4 µmol/L; *p* = 0.001) levels were significantly lower in the case group compared to the healthy control group. Further subgroup analyses among participants with invasive cervical cancer revealed increasing mean levels of MDA (*p* = 0.017) and decreasing mean levels of vitamin C (*p* = 0.004) with increasing stage of the disease. No change in the mean level of glutathione level was observed with each FIGO stage of the disease (*p* = 0.096) (Table 3).

**Figure 1.**
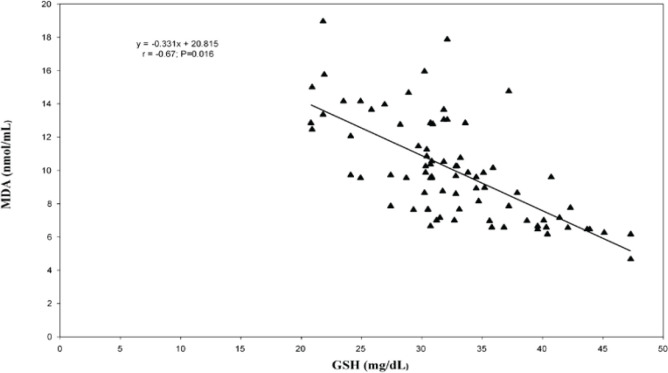
Relationship between erythrocyte reduced glutathione and serum malondialdehyde levels among the cancer patients.

**Figure 2.**
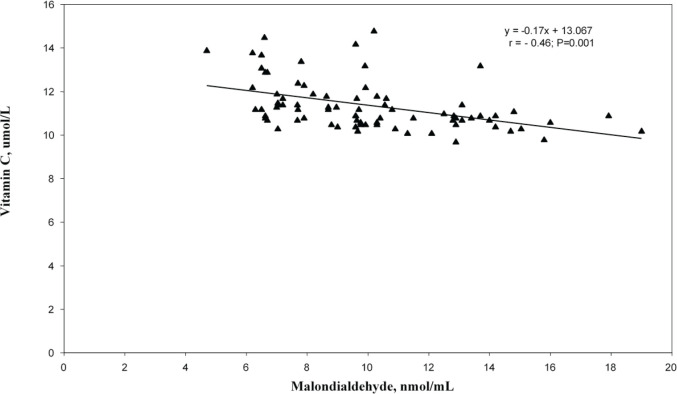
Relationship between serum malondialdehyde and vitamin C levels among the cervical cancer patients.

In the correlation and simple regression analyses, as shown in Figure 1, we found a negative but statistically significant moderately strong association between the mean serum levels of MDA and erythrocyte GSH among women with invasive cervical cancer (*r* = −0.67; *p* = 0.016). We also reported a negative but statistically significant moderately strong association between the mean serum levels of MDA and vitamin C (*r* = −0.46; *p* = 0.001) among these women (Figure 2).

## Discussion

This study investigated the relationship between markers of oxidative stress and cervical cancer and its severity among women in Lagos. The study found a significantly higher level of serum MDA among women with cervical cancer compared to their cancer-free counterparts. We also found a statistically significant reduction in the level of serum vitamin C among those with invasive cervical cancer. The levels of serum MDA increased significantly with the stage of the disease. Inverse relationships were recorded between oxidant (MDA) and antioxidants (GSH and vitamin C) levels in the study, thus indicating a significantly high oxidative stress in participants with invasive cervical cancer.

The progressive increase in the mean MDA levels with the stage of the disease is a pointer to the excessive lipid oxidation and subsequent oxidative stress which occurs with progressive worsening of the disease. This corroborated the findings from previous studies by Naidu et al [4], Nirmala et al [24] and Beevi et al [25]. The significantly lower mean levels of erythrocyte GSH and serum vitamin C found in cervical cancer patients when compared with the cancer-free comparison group may be due to their increased use to scavenge lipid peroxides as well as their sequestration by the tumour cells [11, 13]. This was supported by Nirmala et al [24] who observed significantly lower GSH levels in the erythrocyte and plasma of cervical cancer patients. The mean levels of serum vitamin C showed a statistically significant reduction with the increased stage of the disease, a finding that was in agreement with the study by Naidu et al [4] but differed from the study by Demirci et al [13], which found no significant reduction. The variation in the findings of our current study and that of Demirci et al [13] can be explained by the differences in the baseline characteristics, socio-economic status and nutritional preferences of the participants in both of these studies. The elevated oxidant and reduced antioxidants levels observed among the cervical cancer patients in this study underscore the important roles of oxidative stress in the disease progression. Beevi et al. [25] supported the hypothesis that an imbalance in the oxidant–antioxidant status culminates in lipid oxidative damage, thus potentially providing a mechanistic basis for the initiation and promotion of cancer. A major limitation of this study is the difficulty in excluding women who were unknowingly on vitamin C containing multivitamin supplementations which may have affected the measured serum vitamin C levels in the study. The cross‑sectional design of this study also made it difficult to ascribe any causality based on the associations reported. However, the study has generated hypotheses that can be tested in future well-controlled randomised clinical trials with a large sample size.

## Conclusion

This study has shown that women with invasive cervical cancer have low levels of antioxidants (reduced GSH and vitamin C) and increased MDA, a marker of oxidative stress and these patterns become progressively pronounced with each stage of the disease. The study has, therefore, demonstrated the possible need for antioxidant supplementation in cervical cancer patients to balance their utilisation and reduce oxidative stress which causes damage to various biological molecules that leads to acceleration of the disease stage. More reliable evidence may be obtained from future randomised controlled trials of antioxidant supplementations among cervical cancer patients in Nigeria and Africa where micronutrients deficiencies are still significantly prevalent.

## Conflicts of interest

The authors declared no conflict of interests.

## Funding declaration

This work was supported in part by the Conquer Cancer International Innovation Grant under Project ID 16576 and the Fogarty International Centre of the National Institutes of Health under Award Numbers D43TW010934, D43TW010134 and D43TW010543. The views expressed in this publication are those of the authors and do not necessarily reflect the official views of the American Society of Clinical Oncology® or Conquer Cancer® and the National Institutes of Health.

## Figures and Tables

**Table 1.** Baseline characteristics of the study participants (*n* = 240).^a^

Characteristics	Cases	Controls	*p*-value
	*n* = 120	*n* = 120	
Age (years)	48.0 ± 8.8	46.0 ± 10.7	0.170
Median parity	5.0 (2.0–6.0)	2.0 (0.0–3.0)	0.002
BMI (kg/m^2^)	25.3 ± 5.2	26.4 ± 5.1	0.132
Age at coitarche (years)	19.3 ± 3.4	20.0 ± 3.0	0.129
Age at first delivery (years)	21.1 ± 4.1	26.7 ± 4.5	0.001
Median lifetime sex partners	2.0 (0.0–3.0)	2.0 (0.0–3.0)	0.485
**Educational level**			0.001
Uneducated	16 (20.0)	3 (2.5)	
Primary	26 (32.5)	10 (8.30	
Secondary	23 (28.8)	26 (21.7)	
Post-secondary	15 (18.8)	81 (67.5)	
**Religion**			0.141
Christianity	59 (73.8)	99 (82.5)	
Islam	21 (26.2)	21 (17.5)	
**Marital status**			0.038
Single	1 (1.3)	15 (12.5)	
Married	54 (67.5)	93 (77.5)	
Widowed	25 (31.2)	12 (10.0)	

Abbreviations: BMI, body mass index (calculated as weight in kilograms divided by the square of height in meters).

a Values are given as mean ± SD, median (interquartile range) or number (percentage) unless indicated otherwise.

**Table 2.** Antioxidant and oxidant levels in patients with invasive cervical cancer and their healthy controls (*n* = 240).

Biochemical markers	ICC (*n* = 120)	Controls (*n* = 120)	*p*-value
MDA (nmol/mL)	10.0 ± 3.0	4.4 ± 1.1	0.032
Glutathione (mg/dL)	32.6 ± 6.2	44.2 ± 6.1	0.019
Vitamin C (µmol/L)	12.4 ±2.3	14.6 ± 2.4	0.001

MDA, malondialdehyde; ICC, invasive cervical cancer

**Table 3.** Levels of antioxidants and oxidants for all the FIGO stages of cervical cancer (*n* = 120).

Biochemical markers	FIGO Stages of cervical cancer				*p*-value
	Stage I	Stage II	Stage III	Stage IV	
MDA (nmol/mL)	7.73 ± 1.2	8.47 ± 2.2	10.74 ± 2.4	13.19 ± 2.7	0.017
Glutathione (mg/dL)	35.34 ± 5.0	32.55 ± 8.1	31.69 ± 5.7	30.70 ± 4.8	0.096
Vitamin C (µmol/L)	13.76 ± 2.8	13.49 ± 2.5	11.34 ± 1.2	10.89 ± 0.8	0.004

FIGO, International Federation of Gynaecology and Obstetrics; MDA, malondialdehyde
